# Adequate reimbursement is crucial to support cost-effective rapid on-site cytopathology evaluations

**DOI:** 10.4103/1742-6413.71740

**Published:** 2010-10-18

**Authors:** Mousa A. Al-Abbadi, Leonard I. Bloom, Lisa A. Fatheree, Lori A. Haack, Gerald Minkowitz, David C. Wilbur, Marshall R. Austin

**Affiliations:** James H. Quillen VA Medical Center, Department of Pathology, East Teneesee State University, Johnson City, USA; 1 University of Tennessee Health Science Center, Department of Pathology, Memphis, TN, USA; 2 College of American Pathologists, Northfield, IL, USA; 3 University of Wisconsin Hospital and Clinics, Clinical laboratories, Madison, WI, FL, USA; 4 Minkowitz Pathology, Department of Pathology, Brooklyn, NY, USA; 5 Massachusetts General Hospital, Department of Pathology, Harvard University, Boston, MA, USA; 6 University of Pittsburgh, Pittsburgh, Department of Pathology, PA, USA

Sir,

The majority of us who routinely practice fine needle aspiration biopsy (FNAB) understand the value of immediate on-site evaluation of adequacy/triage and interpretation of this procedure.[1–3] The clinical significance of such evaluations and the critical importance of having a cytopathologist available at the time of the procedure are shared by our nonpathologist colleagues.[4,5] The potential benefits of having cytopathologists evaluating initial FNAB smears and rendering an immediate opinion or preliminary diagnosis increases accuracy and decreases the number of inadequate and suboptimal procedures.[6–11] In one institution, the nondiagnostic rate was reported as 1% for FNABs with on-site evaluations as compared with 20% when on-site evaluation was not provided.[8] The authors estimated that if on-site evaluations were not performed, their direct institutional charges for repeating FNABs would have increased by an additional 2 million US dollars in a 5-year period.[8] Therefore, nondiagnostic FNAB will have two obvious disadvantages: (1) delay in diagnosis, with an obvious negative impact on patient care as well as potential legal consequences and (2) substantial increase in the cost of healthcare.

Since the October 2009 National Coding Corrective Initiative (NCCI) policy manual publication, many of us have faced repeated inquiries about the appropriateness of using the CPT code 88172 pertaining to this immediate cytologic evaluation.[12] The question at hand is whether or not cytopathologists may use multiples of 88172 when remaining on-site for multiple passes by our clinical colleagues. Opinions have surfaced that multiple 88172 codes may not be allowable; some have reported being denied reimbursement for such multiple coding (personal communication). Our billing departments have made inquiries and, despite the fact that there is nothing concrete yet about this issue, the voices demanding clarification are increasing. As with any rejection from a payer, an adequate, accurate and timely appeal may circumvent a discussion pertaining to the propriety of any given procedure, be it 88172 or other. If the payer repeatedly rejects a particular code with a boilerplate denial, further investigation of the insurers’ manual of coding procedures may better direct the appeal process. If all fails, some states have review processes for rejections. This said, as the leading US cytopathology organization, the American Society of Cytopathology (ASC) is joining others in an attempt to express our professional assessment of this matter. Representing the ASC, The Economic and Government Affairs Committee was asked to render a professional opinion through a written editorial commentary on this issue.

The body of evidence is strong that the time required for on-site assessments by cytopathologists is well spent. In a comprehensive cost analysis, Layfield *et al*. found that on-site interpretations consume between 35 and 56 min, exceeding the average time required for frozen section evaluations by approximately 16 min.[8] Not surprisingly, these investigators also found that the Medicare compensation rate for the 88172 code was low in comparison with the other codes performed during a procedure, i.e. 88331, 88332, 88333 and 88334. They calculated that the time costs exceeded compensation by at least $40–50 per case for cytopathologists performing rapid evaluation for adequacy during a procedure. In another extensive cost analysis review of 5,688 FNAB cases, Nasuti *et al*. documented that without on-site evaluation, the rate of inadequate aspirates would increase, resulting in substantial institutional cost for repeat procedures and testing.[9] While billing codes for repeat frozen section procedures (88332) and repeat intraoperative cytologic evaluation (88334) are allowed, it is reasonable to allow similar flexibility for immediate cytologic adequacy evaluations and interpretation when immediate evaluation is required on subsequent cytologic material from the same site, following determination that the prior sample was not adequate for diagnosis.

Given this background and the related questions and uncertainty, it is worth noting that the College of American Pathologists (CAP) recently added a specific question about immediate adequacy assessments and initial interpretations and their documentation in the Laboratory Accreditation Program cytopathology section checklist effective June 2009 (# CYP. 05325). The cytopathology community has the obligation to clearly advocate for reimbursement policies that support cost-effective cytopathology services for patients. It is our hope that this editorial will encourage individual pathologists, our professional organizations and the greater medical community to adequately support cost-effective cytopathology services. Because of widespread confusion on the current correct billing among payers and members, the ASC and CAP submitted a proposed change to the CPT for code 88172 to more clearly define the unit of service.

The Economic and Government Affairs Committee of the American Society of Cytology strongly supports a clear definition of the unit of service for CPT code 88172 that allows the appropriate use of multiple units when multiple procedures are separately evaluated to assure adequacy of the material for diagnosis. As of this writing, the Center for Medicare Services (CMS) is expected to issue additional written guidance on the issue around the November of 2010.

## COMPETING INTEREST STATEMENT BY ALL AUTHORS

No competing interest to declare by any of the authors.

## AUTHORSHIP STATEMENT BY ALL AUTHORS

Each author acknowledges that this final version was read and approved. All authors qualify for authorship as defined by ICMJE http://www.icmje.org/#author Each author participated sufficiently in the work and takes public responsibility for appropriate portions of the content of this article.
